# Regulatory Mechanisms of Mitochondrial Autophagy: Lessons From Yeast

**DOI:** 10.3389/fpls.2019.01479

**Published:** 2019-11-15

**Authors:** Kentaro Furukawa, Aleksei Innokentev, Tomotake Kanki

**Affiliations:** Department of Cellular Physiology, Niigata University Graduate School of Medical and Dental Sciences, Niigata, Japan

**Keywords:** yeast, Atg32, CK2, Ppg1, Far complex, mitochondria, mitophagy

## Abstract

Mitochondria produce the majority of ATP required by cells *via* oxidative phosphorylation. Therefore, regulation of mitochondrial quality and quantity is important for maintaining cellular activities. Mitophagy, the selective degradation of mitochondria, is thought to contribute to control of mitochondrial quality and quantity. In recent years, the molecular mechanism of mitophagy has been extensively studied in yeast and mammalian cells. In particular, identification of the mitophagy receptor Atg32 has contributed to substantial progress in understanding of mitophagy in yeast. This review summarizes the molecular mechanism of mitophagy in yeast and compares it to the mechanism of mitophagy in mammals. We also discuss the current understanding of mitophagy in plants.

## Introduction

Autophagy is a catabolic process that degrades cytoplasmic proteins and organelles. Autophagy induction in yeast results in formation of the pre-autophagosomal structure or phagophore assembly site (PAS), an initial complex of autophagy-related (Atg) proteins, on the vacuole surface. Then, double-membranous structures, termed isolation membranes, emerge from the PAS and extend to sequester cytoplasmic constituents as cargos to form an autophagosome. The autophagosome is then fused to the vacuole, where the cargos are degraded by hydrolytic enzymes for recycling (Nakatogawa et al., 2009). Autophagy was initially thought to be a nonselective degradative process of cytoplasmic constituents. However, recent studies have revealed that autophagy selectively degrades specific cellular components. These include mitochondria, ER, peroxisome, ribosomes, and the cytoplasm-to-vacuole (Cvt) complex [aminopeptidase I (Ape1) and α-mannosidase (Ams1)]. These selective autophagic processes are called mitophagy, ER-phagy, pexophagy, ribophagy, and the Cvt pathway, respectively (Anding and Baehrecke, 2017).

Mitochondria play a pivotal role in cellular activities, including ATP synthesis, calcium buffering, and regulation of apoptosis. During the ATP synthesis process, mitochondria also produce reactive oxygen species (ROS) (Murphy, 2009). Thus, mitochondria are susceptible to oxidative damage. Regulating mitochondria levels to prevent excess ROS production and removing damaged mitochondria to maintain mitochondrial quality are important processes. Mitophagy is thought to be one of the important mechanisms for maintaining mitochondrial homeostasis.

In recent years, the molecular mechanism of mitophagy has been extensively studied in yeast and mammalian cells. Identification of the mitophagy receptor Atg32 has contributed to substantial progress in understanding mitophagy in yeast (Kanki et al., 2009b; Okamoto et al., 2009). However, the molecular mechanism of mitophagy in yeast is not well conserved in mammalian cells and a clear homologue of Atg32 has not been identified. Moreover, there are two types of mitophagy in mammalian cells. One pathway is ubiquitination-mediated and the other is mitophagy receptor-mediated (Pickles et al., 2018).

**Figure 1:**
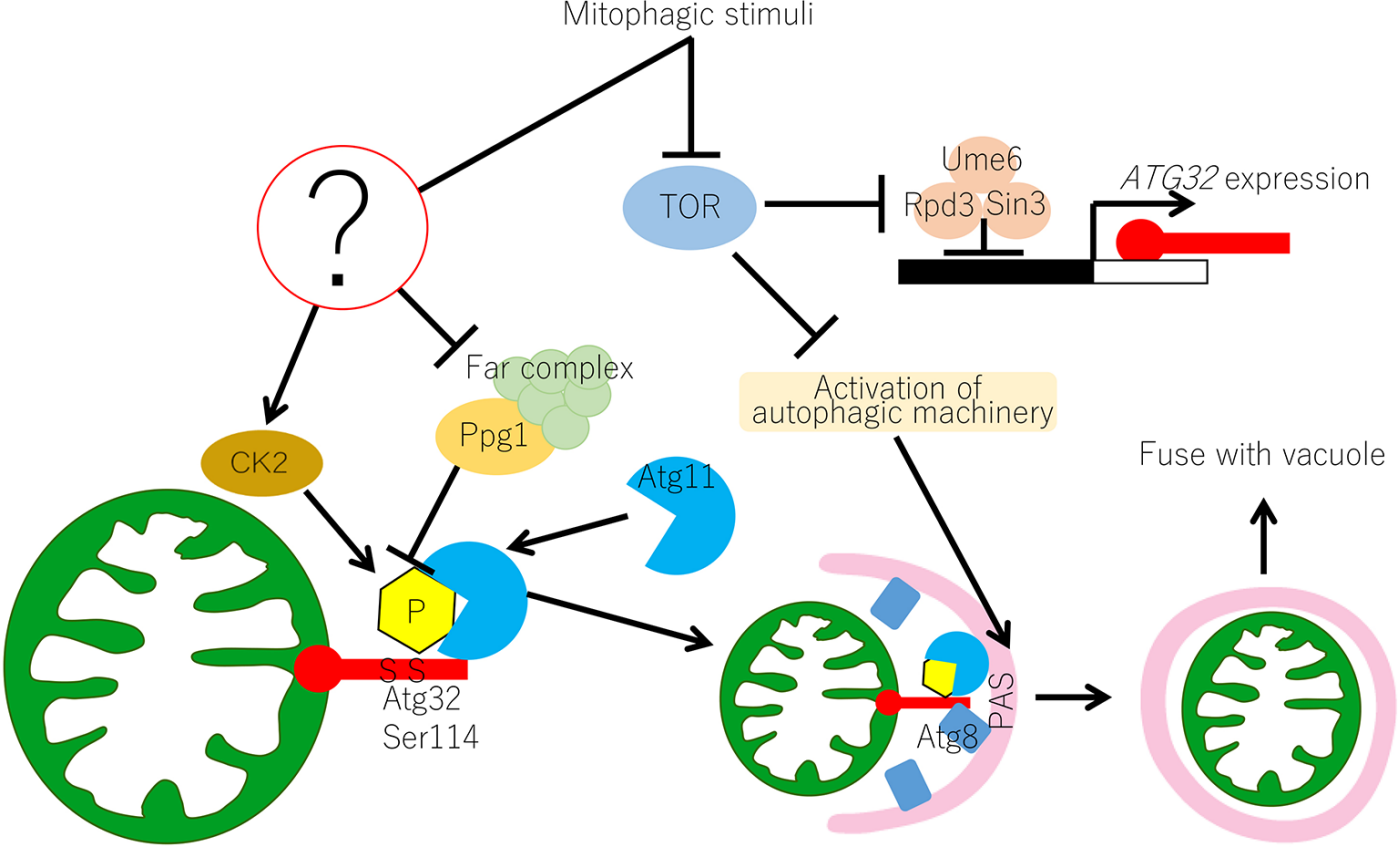
Molecular mechanism of mitophagy in yeast. In yeast, the Atg32-mediated mitophagy is regulated at transcriptional and post-translational levels. Transcription of *ATG32* is suppressed by the Ume6–Sin3–Rpd3 complex, which interacts with the upstream repression sequence (URS) of the *ATG32* promoter region. Inhibition of TOR releases the Ume6–Sin3–Rpd3 complex and *ATG32* can be transcribed. Under normal growing conditions, Ppg1 with the Far complex dephosphorylates Atg32 to prevent unrequired mitophagy. Upon mitophagy induction, the function of the Ppg1-Far complex might be suppressed through unidentified mechanisms and CK2 phosphorylates Atg32 at Ser114 and Ser119. Then, Atg11 interacts with the phosphorylated Atg32 and recruits mitochondria to the PAS. Mitophagy signal also activates the core autophagy machinery, which is recruited to the PAS. At the PAS, Atg32 interacts with Atg8, which anchors on the isolation membrane, and the Atg32–Atg8 interaction facilitates the formation of the autophagosome surrounding the mitochondria. Autophagosome carrying mitochondria eventually fuse with vacuoles for mitochondrial degradation. CK2, casein kinase 2; PAS, phagophore assembly site or pre-autophagosomal structure; TOR, target of rapamycin.

A review of recent progress in the understanding of mitophagy in yeast is presented herein (**Figure 1**). Differences in mitophagy between yeast and mammals are considered. Finally, open questions concerning the molecular mechanism of mitophagy in yeast and the current understanding of mitophagy in plants are discussed.

## Atg11 is an Adaptor Protein for Selective Autophagy in Yeast *Saccharomyces Cerevisiae*

Many of the proteins encoded by autophagy-related (*ATG)* genes that are essential for bulk autophagy are also necessary for selective autophagy (see **Table 1**). In addition, several proteins are specifically required for cargo recognition. One example is the selective adaptor protein, Atg11. Atg11 was first identified as an essential protein for the Cvt pathway, which delivers cytosolic proteins (Ape1 and Ams1) to the vacuole through the autophagy-like pathway (Kim et al., 2001). Atg11 is also required for pexophagy. Atg19 and Atg34 are the adaptor proteins of the Cvt pathway and interact with the Cvt complex (Scott et al., 2001; Suzuki et al., 2010). Atg11 specifically interacts with Atg19 and Atg34, resulting in recruitment of the Cvt complex to the PAS for selective autophagy (Shintani et al., 2002; Suzuki et al., 2010). Similarly, Atg30 in *Pichia pastoris* and Atg36 in *Saccharomyces cerevisiae* are receptor proteins that localize on peroxisomes (Farre et al., 2008; Motley et al., 2012). After induction of pexophagy, Atg11 specifically interacts with Atg30/Atg36 to deliver the peroxisome to the PAS for selective pexophagy. Atg11 is also required for mitophagy and interacts with the mitophagy receptor Atg32. This process is reviewed in the following sections (Kanki and Klionsky, 2008; Kanki et al., 2009b; Okamoto et al., 2009).

**Table 1:** Requirement of *ATG* genes for macroautophagy and mitophagy in *S. cerevisiae*.

ATG Genes	Macroautophagy	Mitophagy
*ATG1*	++	++
*ATG2*	++	++
*ATG3*	++	++
*ATG4*	++	++
*ATG5*	++	++
*ATG6*	++	++
*ATG7*	++	++
*ATG8*	++	++
*ATG9*	++	++
*ATG10*	++	++
*ATG11*	–	++
*ATG12*	++	++
*ATG13*	++	+
*ATG14*	++	++
*ATG15*	(++)	(++)
*ATG16*	++	++
*ATG17*	++	+
*ATG18*	++	++
*ATG19*	−	−
*ATG20*	−	+
*ATG21*	−	+/−
*ATG22*	−	−
*ATG23*	−	+
*ATG24*	−	+
*ATG26*	−	−
*ATG27*	+	+
*ATG29*	++	+/−
*ATG31*	++	+/−
*ATG32*	−	++
*ATG33*	−	+
*ATG34*	−	NA
*ATG36*	−	−
*ATG38*	++	−
*ATG39*	−	−
*ATG40*	−	−
*ATG41*	+	NA
*ATG42*	+	NA

Phenotypes of indicated gene knockout strain: ++, severe defect; +, partial defect; −, no defect. ATG15 encodes a lipase; the cargo can be delivered into the vacuole, but cannot be degraded. ATG19 and ATG34 encode receptors for the Cvt pathway. ATG36 encodes a receptor for pexophagy. ATG39 and ATG40 encode receptors for ER-phagy. ATG25, ATG28, ATG30, and ATG37 are genes related with pexophagy in Pichia pastoris.

## Atg32 IS a Mitophagy Receptor in Yeast

ATG32, the gene that encodes Atg32, was identified by a genome-wide screen of a mutant yeast that displays defective mitophagy. Atg32 is composed of 529 amino acids and has a single transmembrane domain. Atg32 localizes to the mitochondrial outer membrane and its N- and C-terminus are oriented to the cytosol and mitochondrial intermembrane space, respectively. Atg32 works as a mitochondrial receptor protein and interacts with Atg8 and Atg11 (Kanki et al., 2009b; Okamoto et al., 2009).

Atg8 is conjugated to phosphatidylethanolamine by a ubiquitin-like conjugation system and localizes on the isolation membrane (Ichimura et al., 2000). Most of the adaptor and receptor proteins for selective autophagy have a conserved WXXL-like sequence (W/Y-X-X-L/I/V). This sequence is called the Atg8-family interacting motif (AIM) or the LC3-interacting region (LIR) (Noda et al., 2010). Atg8 interacts with adaptor or receptor proteins *via* AIM/LIR to mediate selective recognition of adaptor- or receptor-localizing cargo by the isolation membrane. Atg32 also has an AIM/LIR on its N-terminus and interacts with Atg8 (Okamoto et al., 2009; Kondo-Okamoto et al., 2012). However, Atg32/Atg8 interaction does not play much of a role in mitophagy because an Atg32 mutation in AIM/LIR only partially suppresses mitophagy (Kondo-Okamoto et al., 2012). Atg32/Atg8 interaction may work to extend the isolation membrane along with the mitochondria surface.

Conversely, Atg32/Atg11 interaction plays a crucial role in recognition of mitochondria as cargos. The N-terminus of Atg32 interacts with Atg11 under mitophagy-inducing conditions (Aoki et al., 2011). Atg11 accumulates PAS and tethers the Atg32-localizing mitochondria to the PAS for selective engulfment by the isolation membrane. This Atg32/Atg11 interaction is strictly regulated by the phosphorylation of Atg32 (Aoki et al., 2011).

## Regulation of Mitophagy by Expression and Phosphorylation of Atg32

Mitophagy is efficiently induced when yeast cells are pre-cultured in a non-fermentable medium, then shifted to nitrogen starvation medium containing a fermentable carbon source (Kissova et al., 2004). Atg32 expression is inhibited when cultured in fermentable medium, but is increased in non-fermentable medium or by nitrogen starvation. The conditions that induce Atg32 expression are the same as mitophagy-inducing conditions, suggesting that mitophagy is regulated in part by expression level of Atg32. Atg32 expression is suppressed by the protein kinase TOR and the downstream Ume6–Sin3–Rpd3 complex at the transcription level. Under mitophagy-inducing conditions, such as nitrogen starvation, TOR is suppressed. The Ume6–Sin3–Rpd3 complex then releases its Atg32 transcription repression, resulting in Atg32 expression (Aihara et al., 2014).

Ser-114 and Ser-119 on Atg32 are phosphorylated under mitophagy-inducing conditions. This phosphorylation, especially that of Ser-114 on Atg32, is essential for mitophagy. A Ser to Ala mutation on this residue completely abolishes Atg32/Atg11 interaction and mitophagy. Thus, phosphorylation of Ser-114 on Atg32 is an initial trigger for mitochondrial degradation (Aoki et al., 2011).

An experiment that screened for protein kinase mutants identified casein kinase 2 (CK2) as the kinase that phosphorylates Atg32. Inhibiting CK2 activity using CK2 temperature-sensitive mutants at a non-permissible temperature or using a CK2 inhibitor suppresses Atg32 phosphorylation, Atg32/Atg11 interaction, and mitophagy. Although CK2 is a ubiquitous and constitutively active kinase, Atg32 is not phosphorylated under mitophagy non-inducing conditions (Kanki et al., 2013). This suggests that there is a mechanism that suppresses phosphorylation of Atg32 to prevent unintended loss of mitochondria.

The protein phosphatase 2A (PP2A)-like protein phosphatase Ppg1 was recently identified as a negative regulator of Atg32 phosphorylation (Furukawa et al., 2018). In cells with *ppg1* deletion, Atg32 is constitutively phosphorylated even under mitophagy non-inducing conditions, suggesting that Ppg1 is involved in dephosphorylation of Atg32. Mitophagy is accelerated in *ppg1Δ* cells under specific mitophagy-inducing conditions, further suggesting that Ppg1 contributes to mitophagy inhibition *via* Atg32 dephosphorylation. Generally, the catalytic subunit of PP2A interacts with structural and regulatory subunits. Although Ppg1 is a PP2A family protein, the structural and regulatory subunits of Ppg1 are not well understood. Proteomic analysis showed that Ppg1 co-immunoprecipitates with Far8, one of the Far complex proteins (Far3, Far7, Far8, Far9, Far10, and Far11). Interestingly, cells lacking any of the Far complex components (except Far10) show the same phenotypes as *ppg1Δ* cells, such as Atg32 phosphorylation and accelerated mitophagy. These results suggest that Ppg1 and the Far complex cooperatively dephosphorylate Atg32 and inhibit mitophagy.

Atg32 phosphorylation is presumably regulated by the balance of protein kinase CK2 and protein phosphatase Ppg1. Under mitophagy non-inducing conditions, the Ppg1/Far complex dephosphorylates Atg32 more efficiently than phosphorylation activity of CK2. Conversely, under mitophagy-inducing conditions, CK2 more efficiently phosphorylates Atg32 than dephosphorylation activity of Ppg1.

## Atg32/Atg11 Interaction and Autophagosome Formation Are Minimum Events to Complete Mitophagy

Atg32/Atg11 interaction is a crucial step in mitophagy. Furukawa et al. (2018) found that an Atg32 mutant lacking the 151–200 amino acid region (Atg32Δ151–200) constitutively interacted with Atg11 without any mitophagy-inducing stimuli. Similarly, in cells with ppg1 deletion, Atg32 was constitutively phosphorylated and interacted with Atg11 without any mitophagy-inducing stimuli. However, the Atg32Δ151–200 expressing cells or *ppg1Δ* cells did not induce mitophagy without mitophagy-inducing stimuli, such as nitrogen starvation. Interestingly, mitophagy was induced without any stimuli when the constitutively active form of Atg13 (Atg13-8SA), which activates the autophagy core machinery, was introduced (Kamada et al., 2000; Furukawa et al., 2018). These findings suggest that Atg32/Atg11 interactions and activation of the autophagy core machinery are necessary and sufficient events for mitophagy.

## Regulatory Mechanism of Mitophagy in Yeast

The molecular mechanism of mitophagy in yeast is summarized in **Figure 1**. TOR is inhibited under mitophagy-inducing conditions, such as nitrogen starvation. Downstream of TOR inhibition, the Ume6–Sin3–Rpd3 complex releases transcription suppression of Atg32, resulting in increased Atg32 expression. Atg32 phosphorylation is regulated by the balance of CK2 and Ppg1/Far complex. The Ppg1/Far complex counteracts CK2 and suppresses Atg32 phosphorylation under mitophagy non-inducing conditions, whereas suppression is released and CK2 phosphorylates Atg32 under mitophagy-inducing conditions. It is still unclear how CK2 and Ppg1/Far complex functions are regulated in response to nutrient conditions. Atg11 then interacts with the phosphorylated Atg32 and recruits Atg32 with mitochondria to the PAS. At the PAS, the autophagy core machinery is activated, and the isolation membrane is formed downstream of TOR inhibition. Atg32 interacts with the portion of the isolation membrane where Atg8 is localized. This allows the isolation membrane to extend with the mitochondria surface. Eventually, an autophagosome that completely envelopes the mitochondria is formed.

### Other Factors Affecting Mitophagy

ER-mitochondria contact plays an important role in mitophagy. The ER-mitochondria encounter structure complex (ERMES complex) is a factor that mediates the ER-mitochondria contact site (Lang et al., 2015). Loss of ERMES subunits severely suppresses mitophagy in yeast (Bockler and Westermann, 2014). Loss of ERMES subunits does not affect Atg32/Atg8 interactions, but does affect extension of the isolation membrane. These findings suggest that ER-mitochondria contact is important for lipid supply to promote autophagosome formation during mitophagy. Ubiquitination of ERMES subunits may be a regulatory mechanism of mitophagy, because ubiquitination of the ERMES subunits, Mdm34 and Mdm12, affects mitophagy efficiency (Belgareh-Touze et al., 2017).

Mitochondrial morphology also affects mitophagy efficiency. Mitochondria change their size and morphology by mitochondrial fission and fusion. Because the size of mitochondria under normal culture conditions is typically larger than the autophagosome (∼500 nm diameter), it has been speculated that mitochondrial fission occurs during mitophagy to make mitochondria small fragments which can fit into the autophagosome. Several reports have suggested that mitochondrial fission factors play an important role in mitophagy (Kanki et al., 2009a; Abeliovich et al., 2013; Mao et al., 2013). Mao et al. (2013) reported that Atg11 interacts with mitochondrial fission factor Dnm1 to induce mitochondrial fission for efficient mitophagy. Although mitophagy decreases in cells with deletion of mitochondrial fission factors, such as *dnm1Δ* cells, it is still present at substantial levels (Yamashita et al., 2016). Mendl et al. (2011) reported that mitochondrial fission factors are not required for rapamycin-induced mitophagy. Thus, mitochondrial morphology affects the efficiency of mitophagy, but mitochondrial fission factors are not absolutely essential for mitophagy.

Treatment with the antioxidant N-acetylcysteine (NAC) suppresses mitophagy in yeast cells (Deffieu et al., 2009; Okamoto et al., 2009). In part, this is due to reduced Atg32 expression after NAC treatment. This suggests that oxidative stress is a factor contributing to mitophagy induction.

## Mitophagy in Mammalian Cells

The molecular mechanism of mitophagy in mammalian cells is more complicated than in yeast. There are two independent pathways (Pickles et al., 2018). One pathway is ubiquitination-mediated and the other is mitophagy-receptor-mediated. The ubiquitination-mediated pathway may be related to Parkinson's disease. PTEN-induced putative kinase 1 (PINK1) and Parkin are causative genes of familial Parkinson's disease. Narendra et al. (Narendra et al., 2008) first identified that Parkin, an E3 ubiquitin ligase, accumulates on depolarized mitochondria and induces mitophagy. Subsequent studies revealed that PINK1 is involved in this process (Narendra et al., 2010). PINK1 has a mitochondrial targeting signal (MTS) on its N-terminus. Following PINK1 translation, the N-terminus is constitutively transported into the mitochondrial inner membrane. In the mitochondrial inner membrane, MTS is cleaved and the cleaved PINK1 is released into the cytosol and degraded by the proteasome (Jin et al., 2010; Matsuda et al., 2010; Yamano and Youle, 2013). When mitochondria are damaged and depolarized, the N-terminus of PINK1 cannot translocate to the mitochondrial inner membrane and PINK1 accumulates on the mitochondrial outer membrane. The accumulated PINK1 recruits Parkin from the cytoplasm to the mitochondria, and the Parkin ubiquitinates the mitochondrial outer membrane proteins. Autophagy adaptor proteins such as optineurin (OPTN), neighbor of BRCA1 gene 1 (NBR1), TAX1 binding protein 1 (TAX1BP1), and p62 have a ubiquitin-binding domain and LIR. Thus, these autophagy adaptor proteins connect ubiquitinated mitochondrial proteins and isolation membrane localizing LC3 for selective engulfment of ubiquitinated mitochondria by the autophagosome (Lazarou et al., 2015).

Mitophagy receptor-mediated pathways have some similarities between mammals and yeast. Although an obvious homologue of yeast Atg32 has not been identified, functional counterparts of Atg32 that work as mitophagy receptors have been reported. These include FUN14 domain-containing protein 1 (FUNDC1), BCL2/adenovirus E1B 19-kDa-interacting protein 3 (BNIP3), BNIP3L/Nix, Bcl2-like 13 (Bcl2L13), and FK506 binding protein 8 (FKBP8) (Schweers et al., 2007; Sandoval et al., 2008; Novak et al., 2010; Liu et al., 2012; Murakawa et al., 2015; Bhujabal et al., 2017). All of these receptors are integrated into the mitochondrial outer membrane and have an LIR. The interaction of these receptors and LC3 is the mechanism whereby the isolation membrane identifies the mitochondria as cargos. However, these receptors were identified by different methods. Thus, the importance and distinction of these receptors in several situations and tissues has not been well validated.

## Discussion

This review summarizes the molecular mechanism of mitophagy in yeast (**Figure 1**). The key molecule of mitophagy is the mitophagy receptor protein Atg32. Atg32 phosphorylation is the molecular switch that induces mitophagy. Phosphorylated Atg32 specifically interacts with the adaptor protein Atg11, which recruits Atg32-anchoring mitochondria to the PAS. Atg32 phosphorylation is mediated by CK2 and is suppressed by the Ppg1/Far complex. Future studies should focus on the mechanism that regulates Atg32 phosphorylation. CK2 is a ubiquitous and constitutively active kinase. Thus, there are at least two possibilities for how the Ppg1/Far complex suppresses Atg32 phosphorylation. One possibility is that Ppg1 dephosphorylates Atg32 in opposition to CK2. In this case, Ppg1 activity should be inhibited under mitophagy-inducing conditions. The second possibility is that the Ppg1/Far complex physically interacts with Atg32 to block CK2's access to Atg32. In this case, the Ppg1/Far complex should interact with Atg32 under mitophagy non-inducing conditions and detach from Atg32 under mitophagy-inducing conditions. To date, there is no experimental evidence for direct dephosphorylation of Atg32 by Ppg1 or the interaction of Atg32 and the Ppg1/Far complex. These points need to be investigated by future research.

Another major question remains. In mammalian cells, ubiquitination-mediated mitophagy clearly targets and degrades damaged mitochondria. However, it is not clear whether mitophagy selectively degrades damaged or dysfunctional mitochondria in yeast. It is important to understand whether mitophagy in yeast contributes to mitochondrial quantity control only, or to quality and quantity control. Because mitophagy is completely inhibited in cells with *ATG32* deletion, mitochondria in which phosphorylated Atg32 accumulates should be selected as a cargo. Thus, further understanding of the mechanism of Atg32 phosphorylation and accumulation on specific parts of mitochondrion is necessary to answer this question.

It has been shown that mitophagy also occurs in plants and plays roles in development, stress response, senescence, and programmed cell death (Broda et al., 2018). The link between mitophagy and senescence is best described, but its mechanistic insight is poorly understood. Although core ATG proteins are conserved well in plants and they are required for the senescence-induced breakdown of mitochondria-resident proteins and mitochondrial vesicles (Li et al., 2014; Li and Vierstra, 2014), functional counterparts of mitophagy receptors and PINK1/Parkin mentioned in this review have not been identified in plants. A bioinformatic analysis revealed that *Arabidopsis* has a number of mitochondrial membrane proteins containing ATG8-interacting motifs (Xie et al., 2016), which might act as mitophagy receptors (Broda et al., 2018). To identify mitophagy receptors and reveal regulatory mechanisms of mitophagy in plants, development of research tools such as mitophagy-specific reporters is needed and the lessons from mitophagy/autophagy studies in yeast provide useful insights to those in plants.

## Author Contributions

KF, AI, and TK wrote the manuscript. All authors read and approved the manuscript.

## Funding

This work was supported in part by the Japan Society for the Promotion of Science KAKENHI Grant numbers 19K22419 (TK), 19H05712 (TK), 18H04858 (TK), 18H04691 (TK), 17H03671 (TK), 18K06129 (KF); AMED under Grant Number JP18gm6110013h0001 (TK); Takeda Science Foundation (KF).

## Conflict of Interest

The authors declare that the research was conducted in the absence of any commercial or financial relationships that could be construed as a potential conflict of interest.
